# Trends in hospital admissions and mortality rates for asthma in Ecuador: a joinpoint regression analysis of data from 2000 to 2018

**DOI:** 10.1136/bmjresp-2020-000773

**Published:** 2021-04-30

**Authors:** Angelita Cabrera, Alejandro Rodriguez, Natalia Romero-Sandoval, Sergio Barba, Philip J Cooper

**Affiliations:** 1 Facultad de Ciencias Médicas, Universidad Central del Ecuador, Quito, Pichincha, Ecuador; 2 Universitat de Barcelona, Barcelona, Spain; 3 Escuela de Medicina, Universidad Internacional del Ecuador, Quito, Ecuador; 4 Centro Médico Axxis, Quito, Pichincha, Ecuador; 5 Institute of Infection and Immunity, St George’s University of London, London, UK

**Keywords:** asthma, asthma epidemiology, asthma in primary care

## Abstract

**Background:**

Although asthma has emerged as an important public health problem over recent decades in Latin America, there are limited published data on national hospital admission and mortality rates for asthma from countries in the region.

**Objective:**

To analyse trends in asthma hospitalisation and mortality rates in Ecuador over a 19-year period from 2000 to 2018.

**Methods:**

Hospital discharge and death certificates listing asthma, as defined in the International Classification of Diseases 10th Revision codes (J45 and J46), were used to analyse time trends in rates of hospital admissions and mortality for asthma. The data were obtained from the Ecuadorian National Institute of Statistics and Census. Crude and age-standardised rates were estimated for the entire population. Additionally, specific rates by sex, age and region were estimated. We used joinpoint analysis to identify national trends.

**Result:**

During 2000–2018, a total of 58 250 hospitalisations and 1328 deaths due to asthma were identified. The average annual rates for hospitalisation and mortality attributed to asthma were estimated to be 21 (95% CI 19.3 to 22.8) and 5.2 (95% CI 4.4 to 6.0) per 100 000 population, respectively, over this period. Asthma hospital admissions decreased from 28 to 13.7 per 100 000 population between 2000 and 2018, and asthma mortality decreased from 0.8 to 0.3 per 100 000 population over the same period. Based on jointpoint analysis, two temporal trends were identified for hospital admissions. Between 2000 and 2011, hospital admissions decreased 0.8% per year and between 2011 and 2018 decreased 6.6% per year (p<0.05). On average, hospitalisation rates decreased 3.1% per year (p<0.05) over the entire study period. Mortality rate decreased 5.6% per year (p<0.05) over the 19-year period. Hospitalisation rates were higher among females, those aged 5 to 19 years and those living in the Coast region.

**Conclusions:**

Our analysis shows a temporal trend of reduction in rates of hospitalisations and deaths attributed to asthma between 2000 and 2018 in Ecuador, consistent with similar trends elsewhere in the Latin American region. Health registration systems in Latin America need to be improved to provide reliable data for future between and within country comparisons of trends in asthma hospitalisations and deaths.

**Key messages**

- What are the trends in asthma hospital admissions and mortality rates from 2000 to 2018 in Ecuador by sex, age and region?
- Although a considerable body of literature documenting asthma prevalence and determinants has emerged from Latin America over the past 20 years, there are still limited published data on asthma hospitalisation and mortality rates from countries in the region.
- This study provided, at a national level, a comprehensive perspective on trends and disparities in hospitalisations and mortality related to asthma exacerbations in Ecuador over a 19-year period.

## Introduction

Over recent decades, asthma has emerged as a major challenge for health systems worldwide affecting individuals of all ages.^1^ While the global prevalence of asthma is difficult to estimate with precision because of lack of up-to-date information and data gaps, the most recent global estimates suggest that 339 million people worldwide have asthma.^2^ Asthma causes significant morbidity and direct and indirect costs, especially in low-income and middle-income countries (LMICs).^3^ Poor control of asthma symptoms in LMICs because of inadequate access to health care and medications frequently leads to emergency room visits, hospitalisations and occasionally deaths.

Although hospitals admissions and mortality rates for asthma have decreased worldwide, especially in high-income countries (HICs), >80% of asthma deaths occur in LMICs.^2 3^ In developing regions as Latin America (LA), mortality rates are particularly high among poor marginalised urban populations where underdiagnosis, undertreatment and poor access to healthcare are important issues.^4^ However, although a considerable body of literature documenting asthma prevalence and determinants has emerged from LA over the past 30 or so years,^4 5^ there are still limited published data on asthma hospitalisation and mortality rates from the region. So far, only one study published in 1997 has evaluated asthma mortality rates between different LA countries.^6^ This study showed that, between 1980 and 1990, asthma mortality was high in most of the 11 countries studied.^6^ Recent studies have evaluated trends in asthma hospitalisations and asthma mortality by country: the few such studies from the region are from countries with relatively robust health registration systems such as Brazil, Mexico or Chile.^7–9^

In Ecuador, several studies have been conducted in the last two decades evaluating risk factors for asthma and other allergic diseases.^10–14^ However, little attention has been given to temporal trends on hospitalisations and deaths attributed to asthma. For such a middle-income country, it is important to estimate morbidity and mortality of asthma to provide key information on the health impact and prioritisation of the disease for public health authorities. The present study analysed trends in asthma hospitalisations and asthma mortality rates in Ecuador over a 19-year period between 2000 and 2018.

## Methods

### Study setting

**Figure 1:**
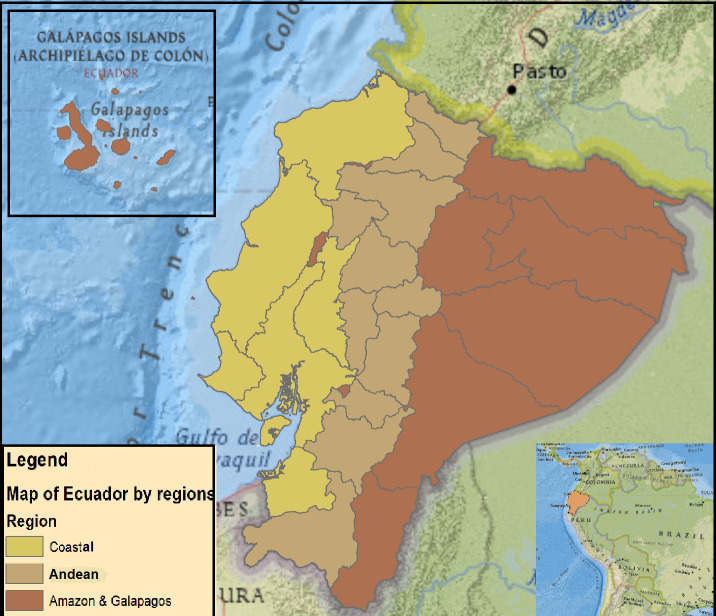
Study area: political map of Ecuador.

Ecuador is an upper-middle-income-country (figure 1) with a per capita income of US$6 080 in 2019.^15^ The country covers a total of 283 560 km^2^ and has four distinct geoclimatic regions: the Andean region that bisects the country North-South consisting of high mountainous terrain reaching an altitude of over 6000 m, interspersed by temperate and subtropical valleys; the Amazon region to the East of the Andes, an area primarily of lowland tropical forest; the Coast region to the west of the Andes, which includes both higher subtropical and lowland tropical areas and the Galapagos Islands approximately 1,000 km to the West of the continent in the Pacific. The population of 17 511, 000 is ethnically diverse: 72% self-identify as Mestizo (mixture of Spanish and indigenous), 7% Indigenous, 6% white, 7% Afro-Ecuadorian and 8% others. Quito and Guayaquil are the largest cities, each with approximately 3 million inhabitants.^16^ Petroleum and agriculture are the principal sources of income with oil accounting for 40% of the country’s exports.^17^ The poverty rate in Ecuador in 2019 was 25%.^15^

Ecuador has a fragmented health system including provision by institutions funded by the government, social security and private sectors.^18^ Public institutions offer healthcare services to the entire population and are divided into four levels of care.^19^ Social security institutions offer health services only to those and their families affiliated through employment. The private sector consists of for-profit entities (hospitals, clinics, dispensaries, doctor’s offices, pharmacies and prepaid medicine companies) and are generally located in the larger cities.^18^ According to the National Institute of Statistics and Censuses (INEC), there were a total of 4168 health institutions in the country in 2017, staffed by 37 293 doctors mostly in the public sector.^20^ Public and private hospitals are required to report health and vital statistics data to the Ministry of Public Health and INEC.

### Study design

We conducted an ecological times-series study to evaluate trends in hospital admissions and mortality rates for asthma in Ecuador. We analysed national databases based on hospitalisation records and death certificates for asthma over the 19-year period from 1 January 2000 to 31 December 2018.

### Data collection

Data were obtained from INEC, the government statistical agency in Ecuador.^16^ INEC compiles data on education, demography, employment, health, households, migration and other population characteristics. Data on hospital admissions and deaths certifications were taken from the Health Statistics section (https://www.ecuadorencifras.gob.ec/documentos/web-inec/Sitios/inec_salud/index.html). We used the International Classification of Diseases 10th Revision codes J45 (asthma, including predominantly allergic asthma, non-allergic asthma, mixed asthma, asthma unspecified) and J46 (status asthmaticus) to identify hospital discharge records and death certificates listing asthma as the primary reason for hospital admission or death. Annual midyear population estimates, total and stratified according to sex, age and region, were obtained from INEC.

### Statistical analysis

Annual crude and age-standardised rates (ASRs) per million population were estimated for the 19-year analysis period. ASRs were calculated using the direct method of standardisation with weights derived from WHO.^21^ Additionally, crude rates by age group (≤5; 5–19; 20–44; 45–64; >64 years), sex and region (Coast, Andean and rest of the country) were estimated for hospitalisation and mortality data.

Joinpoint regression models were used to evaluate trends in asthma hospital admissions and mortality rates over the study period.^22^ This method identifies the year(s) when a trend change is produced by connecting several different line segments on a log scale at ‘joinpoints’. The analysis starts with zero joinpoints (ie, a straight line) and then identifies points where a statistically significant change over time in the linear slope of the trend occurred, adding these points to the model. Joinpoint method provides the Annual Percentage Change (APC) in rates between trend-change points, and estimates also the Average Annual Percentage Change (AAPC) over the whole study period. The APC is tested to determine if it differs from that expected under the null hypothesis (ie, APC is 0%). In the final model, each joinpoint indicates a statistically significant change in trends (increase or decrease) and each of those trends is described by an APC. When there are no joinpoints (ie, no changes in trend), APC is constant and equals AAPC.

We conducted joinpoint analysis for the total population and stratified by age group, sex and region using crude hospitalisation and mortality rates. APC and AAPC were calculated with 95% CI. P value <0.05 was considered statistically significant. Asthma hospital admissions and mortality rates were estimated using Epidat (V.4.2) and joinpoint analyses using the Joinpoint software (V.4.8.0.1) of the Surveillance Research Programme of the US National Cancer Institute.^23^

### Patient and public involvement

This analysis was based on anonymised secondary data from hospital discharge records and death certificates.

## Results

### General characteristics of asthma hospital admissions and asthma mortality rates

Table 1 shows numbers and crude average annual rates for hospitalisations and deaths attributed to asthma for the total population and by variables of interest. In the case of hospital admissions, from 2000 to 2018, a total of 58 250 asthma hospital discharge records were identified in INEC databases. Of these, 31 227 (53.4%) were for females, 20 605 (35.4%) were for 5–19 years and 31 597 (54.2%) were from the Coast region. The average annual rate of asthma hospitalisations over the 19-year study period was 21 (95% CI 19.3 to 22.8) admissions per 100 000 population. Average annual hospitalisation rates were highest among children below 5 years and in the Amazon region and Galapagos Islands.

**Table 1:** Number and average annual rates for asthma hospital admissions and mortality in Ecuador, 2000–2018

Characteristics	Hospital admissions for asthma n (%)	Average annual rate of hospital admissions per 100 000 population (95% CI)	Asthma deaths n (%)	Average asthma mortality rate per 100 000 population (95% CI)
Overall	58 250 (100)	21 (19.3 to 22.8)	1328 (100)	0.5 (0.44 to 0.6)
Sex				
Male	27 123 (46.6)	19.6 (18 to 21.2)	663 (49.9)	0.52 (0.43 to 0.59)
Female	31 227 (53.4)	22.6 (20.5 to 24.7)	665 (50.1)	0.52 (0.42 to 0.61)
Age group (years)				
<5	16 087 (27.6)	51.9 (47.5 to 56.3)	128 (9)	0.44 (0.27 to 0.57)
5–19	20 605 (35.4)	23.8 (22.4 to 25.1)	122 (8.6)	0.13 (0.09 to 0.18)
20–44	8582 (14.7)	8.5 (7.4 to 9.5)	151 (10.6)	0.14 (0.11 to 0.17)
45–64	6852 (11.8)	16.9 (14 to 19.7)	178 (12.5)	0.42 (0.33 to 0.51)
>64	6119 (10.5)	37.6 (29.9 to 45.3)	843 (59.3)	4.9 (4.13 to 5.80)
Region				
Coast	31 597 (54.2)	22.8 (20.6 to 25.1)	782 (58.9)	0.59 (0.53 to 0.67)
Andean	21 967 (37.8)	17.7 (16.5 to 19)	512 (38.5)	0.46 (0.32 to 0.59)
Amazon and Galapagos	4500 (8)	32.4 (28 to 36.9)	34 (2.60)	0.27 (0.12 to 0.43)

A total of 1328 deaths were identified over the 19-year study period in Ecuador, of which 665 (50.1%) were females, 843 (59.3%) were aged >64 years and 782 (58.9%) were living in the Coast region. The average annual asthma mortality rate was 0.5 (95% CI 0.44 to 0.6) deaths per 100 000 population. Significantly higher mortality rates were observed among the population aged >64 years.

### Trend analysis

Table 2 shows numbers and rates by year for asthma hospitalisations for the whole population and by age, sex and region. Trend analysis in the whole population showed that crude and adjusted asthma hospitalisation rates decreased. ASRs decreased from 27.8 to 12.9 admissions per 100 000 population between 2000 and 2018. Similarly, significantly decreasing trends were observed for hospital admission rates by sex, age and region over the study period (see table 2). Table 3 shows numbers and asthma mortality rates by year for the total population and by age, sex and region. Crude and adjusted mortality rates decreased in the study period. ASRs decreased from 1.1 to 0.4 per 100 000 population between 2000 and 2018. Significantly decreasing trends were also observed for asthma mortality rates by sex, age and region (see table 3).

**Table 2:** Crude rates, ASRs and group annual rates for asthma hospital admissions per 100 000 population, Ecuador 2000–2018

Year	Hospital admissions			Age groups (years)					Sex		Region		
	N	Crude	ASRs	<5	5–19	20–44	45–64	>64	Male	Female	Coast	Andean	AG
2000	3513	28	27.8	62.8	28.2	11.9	28.7	60.9	25.3	30.8	29.6	24.1	49.1
2001	2812	21.9	22.2	45.6	21.3	10.1	21.9	57.4	18.8	25.1	22.8	18.9	40
2002	2919	22.3	22.0	50.1	21.4	10.7	21.1	50.1	19.7	26.6	23.3	18.5	44
2003	2933	22.0	21.6	55.3	20.1	9.2	21.7	49.2	19.4	24.8	22.4	19.5	40.2
2004	3208	23.7	23.3	61.6	19.2	10.4	24.6	55.3	21.3	26.1	25.0	19.9	43.6
2005	3409	24.8	24.0	61.7	23.6	11.1	19.4	59.0	23.2	26.5	26.2	21.0	43.7
2006	3057	21.9	21.4	49.6	20.6	10.5	20.9	47.6	19.9	23.9	22.9	19.3	33.6
2007	3381	23.8	22.4	62.1	24.9	9.1	18.5	46.7	22.5	25.0	27.7	18.5	31.5
2008	3574	24.7	23.2	62.5	25.9	10.2	20.4	43.9	24.1	25.3	28.4	19.6	33.3
2009	3277	22.2	21.0	53.7	23.8	9.2	18.9	40.3	20.6	23.9	25.4	17.1	36.7
2010	3344	22.3	20.6	53.2	27.0	9.1	15.7	35.0	21.2	23.3	25.7	17.7	28.7
2011	3320	21.7	19.9	58.2	26.7	7.3	15.5	30.9	20.7	22.8	26.5	15.5	29.0
2012	3207	20.7	18.9	54.4	26.4	8.2	13.4	24.2	20.0	21.3	23.5	16.3	31.8
2013	3103	19.6	18	48.7	26.5	8.0	11.9	24.0	18.3	21.0	22.9	15.7	23.5
2014	3092	19.3	17.6	53.9	27.0	6.1	11.5	21.2	18.9	19.7	21.4	16.4	24.0
2015	2657	16.3	15	44.0	23.3	5.4	9.8	18.3	16.0	16.6	16.8	14.8	24.6
2016	2676	16.2	14.9	43.7	23.9	4.9	9.6	18.5	15.4	16.9	15.8	16.0	21.3
2017	2435	14.5	13.6	32.4	22.2	5.6	8.7	17.5	13.7	15.4	14.1	14.3	20.4
2018	2333	13.7	12.9	33.7	20.6	5.1	8.7	14.6	13.0	14.4	13.6	13.5	17.1

AG, Amazon and Galapagos regions; ASR, age-standardised rate.

**Table 3:** Asthma mortality rates by age groups, sex and regions per 100 000 population

Year	Asthma mortality			Age groups (years)					Sex		Region		
	N	Crude	ASRs	0–4	5–19	20–44	45–64	>64	Male	Female	Coast	Andean	AG
2000	106	0.8	1.1	1.1	0.24	0.32	0.73	8.3	0.72	0.98	0.89	0.85	0.35
2001	87	0.7	0.9	1.1	0.22	0.10	0.70	6.8	0.67	0.69	0.51	0.80	1.34
2002	85	0.6	0.8	0.8	0.29	0.23	0.39	5.9	0.70	0.60	0.53	0.82	0.33
2003	77	0.6	0.7	0.6	0.21	0.14	0.54	5.5	0.63	0.52	0.49	0.72	0.16
2004	86	0.6	0.7	0.8	0.23	0.22	0.52	5.6	0.66	0.60	0.56	0.73	0.61
2005	93	0.7	0.8	0.4	0.28	0.14	0.62	6.9	0.57	0.78	0.77	0.63	0.15
2006	83	0.6	0.7	0.2	0.14	0.23	0.35	6.5	0.69	0.50	0.66	0.59	0.00
2007	90	0.6	0.8	0.5	0.13	0.15	0.77	6.0	0.55	0.71	0.66	0.66	0.14
2008	88	0.6	0.7	0.5	0.16	0.17	0.28	6.3	0.68	0.54	0.79	0.45	0.27
2009	72	0.5	0.6	0.2	0.09	0.13	0.36	5.3	0.49	0.49	0.62	0.38	0.13
2010	78	0.5	0.6	0.5	0.07	0.17	0.60	4.4	0.54	0.50	0.72	0.30	0.51
2011	71	0.5	0.5	0.1	0.11	0.11	0.33	5.0	0.48	0.45	0.66	0.28	0.25
2012	54	0.3	0.4	0.2	0.08	0.11	0.24	3.3	0.35	0.34	0.58	0.12	0.12
2013	58	0.4	0.4	0.2	0.00	0.09	0.43	3.7	0.32	0.41	0.55	0.18	0.24
2014	67	0.4	0.5	0.4	0.17	0.09	0.31	3.7	0.40	0.43	0.60	0.24	0.23
2015	50	0.3	0.3	0.1	0.10	0.05	0.22	3.0	0.35	0.27	0.41	0.23	0.00
2016	46	0.3	0.3	0.1	0.04	0.10	0.25	2.5	0.26	0.30	0.45	0.11	0.11
2017	37	0.2	0.2	0.2	0.06	0.07	0.14	2.0	0.23	0.22	0.34	0.12	0.00
2018	54	0.3	0.4	0.1	0.00	0.14	0.24	3.0	0.30	0.34	0.51	0.25	0.11

AG, Amazon and Galapagos regions; ASR, age-standardised rate.

### Joinpoint analysis

**Figure 2:**
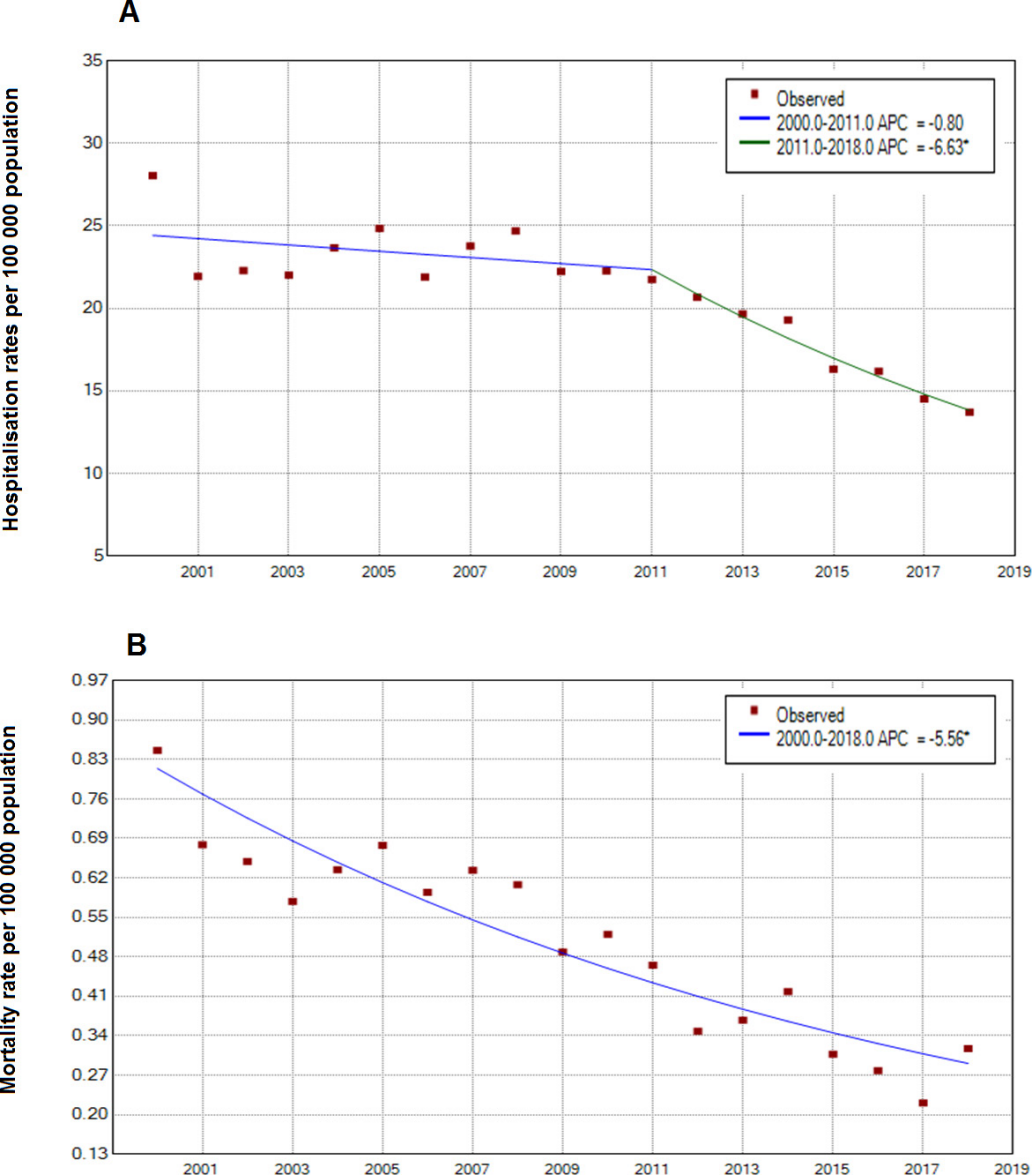
National trends in asthma hospital admissions and asthma mortality in Ecuador, period 2000–2018. APC, Annual Percentage Change.

**Figure 3:**
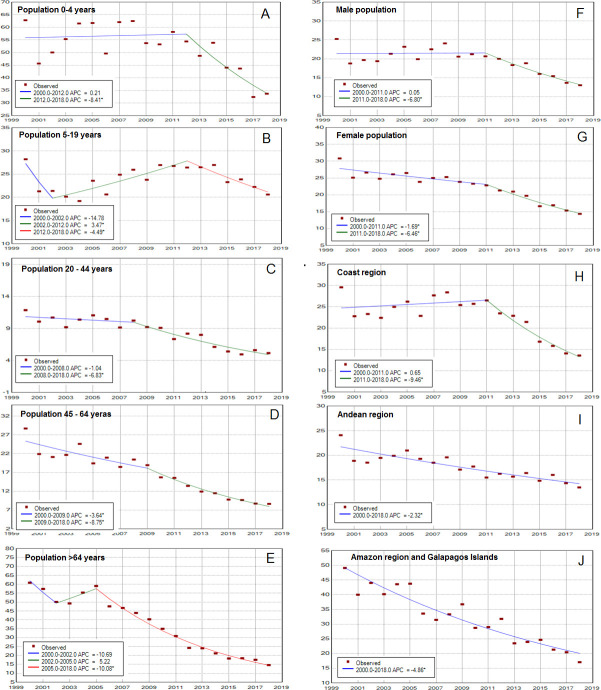
Trends in asthma hospital admissions per 100 000 population by age, sex and region, period 2000–2018. (A) Population aged 0-4 years; (B) population aged 5–19 years; (C) population aged 20–44 years; (D) population aged 45–64 years; (E) population aged >64 years; (F) male population; (G) female population; (H) population residing in the Coast region; (I) population residing in the Andean region; (J) population residing in the Amazon region and Galapagos Islands. APC, Annual Percentage Change.

Figure 2 and table 4 show joinpoint analysis for crude asthma hospitalisation and mortality rates. For the whole population, hospital admissions decreased 0.8% per year from 2000 to 2011 and 6.6% per year from 2011 to 2018 (p<0.05) (figure 2). On average (AAPC), hospitalisation rates decreased 3.1% per year over the study period (table 4). Similarly, joinpoint analysis identified several trends in hospital admissions for crude rates by sex, age and region (see figure 3). For age groups of 0–4, 5–19, 20–44, 45–64 and >64 years, the AAPC decreased 2.7%, 1.4%, 4.3%, 6.2% and 7.88% per year, respectively, over the study period (figure 3A–E). For the male and female populations, the AAPC decreased 2.7% and 3.6% per year, respectively, over the study period (figure 3F, G). By region, the AAPC decreased 3.4% per year in the Coast region, 2.3% in the Andean region and 4.9% in the Amazon and Galapagos region, over the study period (figure 3H–J).

**Table 4:** Joinpoint analysis for crude asthma hospital admissions and mortality rates in Ecuador, 2000–2018

	Full range 2000 - 2018	Trend 1		Trend 2		Trend 3	
	AAPC (95% CI)	Years	APC (95% CI)	Years	APC (95% CI)	Years	APC (95% CI)
Asthma Hospital Admissions							
Total sample	-3.1* (-4.3 to -1.9)	2000–2011	-0.8 (-2.1 to 0.5)	2011–2018	-6.6 * (-9.3 to -3.9)		
Sex							
Male	-2.7* (-4.2 to -1.1)	2000–2011	0.0 (-1.6 to 1.8)	2011–2018	-6.8* (-10.1 to -3.4)		
Female	-3.6 (-4.5 to -2.6)	2000–2011	-1.7* (-2.7 to -0.7)	2011–2018	-6.5* (-8.5 to -4.4)		
Age group							
<5	-2.7* (-4.9 to -0.6)	2000–2012	0.2 (-1.6 to 2.1)	2012–2018	-8.4* (-13.8 to -2.6)		
5-19	-1.4 (-3.6 to 0.9)	2000–2002	-14.8 (-29.6 to 3.2)	2002–2012	3.5* (1.7 to 5.3)	2012 - 2018	-4.5* (-7.3 to -1.6)
20-44	-4.3* (-5.8 to -2.7)	2000–2008	-1 (-3.8 to 1.7)	2008–2018	-6.8* (-8.9 to -4.7)		
45-64	-6.2* (-7.6 to -4.8)	2000–2009	-3.6* (-5.7 to -1.6)	2009–2018	-8.7* (-11 to -6.4)		
>64	-7.8* (-10.6 to -4.8)	2000–2002	-10.7 (-25 to 6.4)	2002 – 2005	5.2 (-11.5 to 25.1)	2005 - 2018	-10.1* (-11 to - 9.1)
Region							
Coast	-3.4* (-5 to -1.8)	2000–2011	0.7 (-1.1 to 2.4)	2011–2018	-9.5* (-12.8 to -6)		
Andean	-2.3* (-2.9 to -1.7)	2000–2018	-2.3* (-2.9 to -1.7)				
Amazon	-4.9* (-5.7 to -4)	2000–2018	-4.9* (-5.7 to -4)				
Asthma Mortality							
Total sample	-5.6* (-6.7 to -4.4)	2000–2018	-5.6* (-6.7 to -4.4)				
Sex							
Male	-5.6* (-7.5 to -3.7)	2000–2008	-1.9 (-5.2 to 1.4)	2008–2018	-8.5* (-11.1 to -5.8)		
Female	-5.7* (-7.2 to -4.1)	2000–2018	-5.7* (-7.2 to -4.1)				
Age group							
<5	-11.1* (-14 to -8.1)	2000–2018	-11.1* (-14 to -8.1)				
5-19	–						
20-44	-5.6* (-8.2 to -2.9)	2000–2018	-5.6* (-8.2 to -2.9)				
45-64	-6* (-8.7 to -3.3)	2000–2018	-6* (-8.7 to -3.3)				
>64	-5.8* (-7.2 to -4.4)	2000–2018	-5.8* (-7.2 to -4.4)				
Region							
Coast	-3.8 (-9.3 to 2)	2000–2002	-22.4 (-51.3to 23.6)	2002–2008	7.3 (-3.6 to 19.3)	2008 - 2018	-5.9* (-9.2 to -2.5)
Andean	-9.7* (-11.7 to -7.7)	2000–2018	-9.7* (-11.7 to -7.7)				
Amazon							

*P<0.05.

AAPC, Average Annual Percentage Change; APC, Annual Percentage Change.

**Figure 4:**
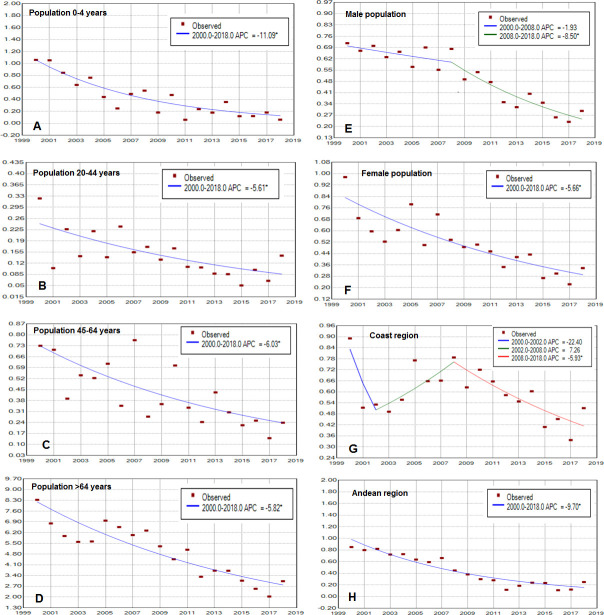
Trends in asthma mortality rate per 100 000 population by age, sex and region, period 2000–2018. (A) Population aged 0–4 years; (B) population aged 20–44 years; (C) population aged 45–64 years; (D) population aged >64 years; (E) male population; (F) female population; (G) population residing in the Coast region; (H) population residing in the Andean region. APC, Annual Percentage Change.

Figure 2 and table 4 show joinpoint analysis for crude asthma mortality rates. For the overall population, deaths decreased 5.6% per year (p<0.05) over the 19-year period (figure 2). Similarly, joinpoint analysis identified several trends in asthma mortality for crude rates by sex, age and region (see figure 4). For age groups of 0–4, 20–44, 45–64 and >64 years, the AAPC decreased 11.1%, 5.6%, 6% and 5.8% per year, respectively, over the study period (figure 4A–D). For the male and female populations, the AAPC decreased 5.6% and 5.7% per year, respectively, over the study period (figure 4E, F). By region, the AAPC decreased 3.8% per year in the Coast region and 9.7% per year in the Andean region (figure 4G, H). Jointpoint analysis technique is unable to work with zeros, so models for the population aged 5–19 years and for the population residing in the Amazon region and Galapagos Islands were not calculated due to these groups presenting zero deaths in some years of the study period (see table 3).

## Discussion

To our knowledge, this is the first study from Ecuador to evaluate changes over time of hospital admissions and mortality from asthma. We did a time trend analysis to describe changes over a 19-year period (between 2000 and 2018) in asthma hospitalisation and mortality rates using data from national hospital discharge records and death certificates. Our results showed significant reductions in asthma hospital admission and mortality rates in Ecuador over this period. Decreasing rates were observed for both sexes, and across all age groups and geo-climatic regions of the country, although the magnitude of these declines varied greatly by the factor studied with greatest reductions seen among the younger and older age groups (ie, <5 and >64 years), in the Coast region and among men.

Prior to 1980, asthma morbidity and mortality rates were seen to increase in HICs, leading to the suggestion of an asthma epidemic, although such trends appeared to stabilise or even decline after the 1980s.^24 25^ More limited data, however, have been available on asthma morbidity and mortality from LMICs. In the case of asthma mortality, several studies have been done in LA prior to 2000 showing high mortality rates for asthma during the 1980s and a trend of decreasing mortality up to 2000.^6 26–29^ A study evaluating data for 11 countries in the region between 1980 and 1992 showed that Uruguay and Mexico had the highest mortality rates (both with 5.6 asthma deaths per million population) and Paraguay and Colombia had the lowest rates (0.8 and 1.35 per million, respectively).^6^ Recent studies using data for the last 20 years have shown that asthma mortality continues to decrease in the region among all age groups.^8 30–33^ For example, data from Costa Rica, Mexico, Brazil and Puerto Rico showed decreasing death rates of 80%, 26%, 10% and 42%, respectively.^8 30 34 35^ An study using data from WHO of 46 countries also showed trends of decreasing asthma mortality, as did studies conducted in Kuwait and the USA.^25 36 37^ Consistent with these findings, our data showed a reduction in crude asthma mortality rates of 5.6% per year between 2000 and 2018. The tendency of decreasing mortality rates was most pronounced in young children (<5 years of age), among whom a decrease of 11.1% per year was observed over the study period. Similarly, national death rates in Brazil among children declined by 87% between 1980 and 2014.^34^

Analysis of time trends for asthma hospitalisations is more problematic, particularly for between-country comparisons, because of limitations in registration systems.^2^ Despite potential limitations, hospitalisation rates are frequently used as as an indicator for improvements in asthma care and burden of morbidity, as well as asthma severity and disease control.^1^ Of studies from HICs where registration data for asthma hospital admissions are easily available, almost all have shown trends of reductions in hospitalisation rates over time over the last three decades, particularly in European countries.^2^ Studies conducted in Kuwait (period 2000–2014) and Costa Rica (period 2000–2011) also showed a reduction in asthma hospital admissions rates of 16% and 53%, respectively.^35 36^ A study from Brazil showed a 36% decrease in the absolute number of hospital admissions between 2008 and 2013,^34^ while in Chile (period 2000–2014) and Israel (period 1990–1999), the number of hospital admissions increased.^38 39^ Our data showed a decline of 6.6% per year in asthma hospitaliations between 2011 and 2018, which was even greater among those aged >64 years.

We found important differences in asthma hospitalisation and mortality rates by age and between different regions of Ecuador. The average annual rate of hospitalisations in young children was 2–6 times greater compared to older age groups. This could be accounted for by asthma attacks caused by respiratory viral infections which affect younger children most.^40^ Populations living in Coast and Amazon regions accounted for 62% of hospital admissions over the 19-year observation period and showed higher hospitalisation rates compared with the Andean population. Such a difference could be accounted for by climatic factors (ie, predominantly tropical vs temperate climates) that could alter patterns of risk factors for asthma attacks (eg, exposures to aeroallergens). Interestingly, the phase I and III studies of the International Study of Asthma and Allergies in Childhood done in South American centres showed a pattern of higher asthma prevalence among children living in coastal cities with tropical climates.^41^ In this study, asthma mortality rates were similar among males and females, an observation that runs contrary to other epidemiological data: asthma prevalence, severity, exacerbation rates, hospitalisations and mortality are higher among women than men.^42^ A possible explanation could be a cultural factor: men are less likely to visit health services compared to women. This might explain a greater number ofasthma hospitalisations, but, because of the healthcare provided, a similar number of asthma-related deaths among women compared to men.

In the joinpoint analysis of asthma hospitalisation rates, we observed that rates decreased from 2011 for several of the categories for age, sex and region. This is unlikely to be explained by changes in registration methodology (ie, not consistent across all categories), and might reflect trends of decreasing hospitalisation rates related to systematic changes in national management strategies and access to asthma medications. In Brazil, for example, a programme to manage and control asthma was implemented in the city of Salvador (ProAR), leading to a marked reduction in asthma hospitalisations.^43^ In Ecuador, a programme focused in reductions in the number of ambulatory hospitalisations for children <5 years called Atencion Integrada a las enfermedades prevalentes de la Infancia could have contributed to decreased hospitalisations and deaths. Other factors that might have contributed to declines in asthma hospitalisation as well as death rates in Ecuador are the improvements in the national health system through policies of inclusion in social and public health programmes since 2005.^44 45^ Additionally, although the country does not have national guidelines for asthma management, the widespread use of international guidelines such as those of the Global Initiative for Asthma could have contributed to improved asthma management and control nationally.

Our study has a number of important limitations. First, although the Ecuadorian registration system for deaths and hospital admissions has improved over the last decade, under-reporting (and under-recognition of asthma) remains an issue. However, this is unlikely to fully account for the observed long-term trends. Second, there are potential diagnostic limitations and imprecise data at the time death certificates are completed, especially in cases where ‘non-specific’ respiratory failure has been listed as the underlying cause of death. Third, diagnostic issues with respect to asthma are more common in children <4 years of age due to similarities with other presentations of respiratory disease. Diagnostic procedures such as spirometry are unsuitable in young children. Finally, we do not have data relating to hospitalisations such as disease severity, presence of comorbidities, adherence to maintenance treatment or therapy received.

## Conclusions

Here, we have done a joinpoint analysis of national data from Ecuador for asthma hospitalisation and death rates over a 19-year period between 2000 and 2018. Our data show temporal trends of reductions in hospitalisation and death rates attributed to asthma and are generally consistent with data published elsewhere in the Latin American region. Trends of decreasing rates were observed for both sexes, all age groups and in all geo-climatic regions of the country. Future studies, evaluating potential links between asthma hospitalisations/deaths and health and social inclusion programmes as well as specific socioeconomic indicators, could provide insights into potential factors that underlie these national trends. Improvements are required to national health registries in the LA region to improve the quality and quantity of information available on hospital admissions and mortality. We need to understand better how research using health registries can be used to improve health outcomes, especially in LMICs. Clinical data registries are extremely valuable when their use can be shown to improve quality of care and population health.

## Data Availability

All data relevant to the study are included in the article or uploaded as supplementary information.
